# *In vitro* activity of imipenem–relebactam against carbapenem-resistant *Pseudomonas aeruginosa*: correlation with carbapenem MICs and association with *β*-lactamase classes

**DOI:** 10.3389/fmicb.2026.1790477

**Published:** 2026-05-07

**Authors:** Aynaa I. Alsharidi, Rand A. Alhumaidi, Ahmed M. Albarrag, Jaffar A. Al-Tawfiq, Ali M. Somily

**Affiliations:** 1 Division of Infectious Diseases, Department of Medicine, College of Medicine, King Saud University, Riyadh, Saudi Arabia; 2 Department of Pathology (Microbiology Unit), College of Medicine, King Saud University Medical City (KSUMC), King Saud University (KSU), Riyadh, Saudi Arabia; 3 Infectious Disease Unit, Specialty Internal Medicine, Johns Hopkins Aramco Healthcare, Dhahran, Saudi Arabia; 4 Division of Infectious Diseases, Indiana University School of Medicine, Indianapolis, IN, United States; 5 Division of Infectious Diseases, Johns Hopkins University, Baltimore, MD, United States; 6 Accreditation and Infection Control Division, Quality and Patient Safety Department, Johns Hopkins Aramco Healthcare, Dhahran, Saudi Arabia

**Keywords:** antimicrobial resistance, carbapenemases, Carbapenem-resistant *Pseudomonas aeruginosa*, imipenem-relebactam, *in vitro* activity, metallo-*β*-lactamases, sequence types, WGS

## Abstract

**Introduction:**

Carbapenem-resistant *P. aeruginosa* (CRPA) poses a major therapeutic challenge due to the limited number of available treatment options. Imipenem-relebactam (IMP-REL), a carbapenem combined with a Class A/C *β*-lactamase inhibitor, has demonstrated activity against selected multidrug-resistant isolates. This retrospective, observational, analytical, laboratory-based study represents one of the early reports from Saudi Arabia and the Middle East and North Africa (MENA) region evaluating the *in vitro* activity of IMP-REL using archived clinical isolates with prospectively performed testing, and examining its association with carbapenem minimum inhibitory concentrations (MICs) and *β*-lactamase classes.

**Methods:**

Ninety-nine CRPA isolates were subjected to antimicrobial susceptibility testing, and 66 isolates were selected randomly for whole-genome sequencing. The *E*-test is used to determine MICs for imipenem, meropenem, and IMP-REL. Correlation analysis, regression modeling, MIC_50_/MIC_90_ computation, and comparisons of *β*-lactamase class subgroups were performed. Scatter plots and MIC distribution curves were generated to visualize these associations.

**Results:**

IMP-REL MIC values were available for the 99 non-duplicate CRPA isolates. Most isolates originated from respiratory specimens (51%), followed by body fluids (18%) and urine (10%). IMP-REL MICs ranged from 0.38 to 32 mg/L, with a median MIC of 1.5 mg/L and MIC₅₀/₉₀ values of 1.5 and 12 mg/L, respectively. Based on CLSI criteria, 78.8% of isolates were susceptible, 10.1% intermediate, and 11.1% resistant. MIC distributions demonstrated clustering at low concentrations with a distinct resistant tail. Weak but statistically significant positive rank-based correlations were observed between IMP-REL MICs and imipenem (*ρ* = 0.21, *p* = 0.041) and meropenem MICs (*ρ* = 0.27, *p* = 0.0077). Among the 66 isolates with characterized resistance mechanisms, Class C and Class D *β*-lactamases and efflux pumps were universally present, while Class A (13.6%) and Class B (7.6%) β-lactamases were infrequent.

**Conclusion:**

IMP-REL shows substantial *in vitro* activity against CRPA, despite only weak correlations with carbapenem MICs and no meaningful association with predominant Class C and Class D *β*-lactamases. These findings support IMP-REL as an important therapeutic option for CRPA infections in settings with high antimicrobial resistance.

## 1 Introduction

*Pseudomonas aeruginosa* is a major cause of severe hospital-acquired infections. Its intrinsic resistance—including efflux pump overexpression, reduced permeability, and *β*-lactamase production—poses significant challenges to clinical management (Pang et al., 2019).

Carbapenem-resistant *P. aeruginosa* (CRPA) represents a global public health threat, with reported prevalence of carbapenem resistance among *P. aeruginosa* isolates varying by region, ranging from approximately 30% in the Middle East to 69% in parts of Central America (Reyes et al., 2023). CRPA bloodstream infection-associated 30-day mortality has been reported to reach up to 30% (Reyes et al., 2023). Accordingly, CRPA is classified as a high-priority pathogen in the World Health Organization (WHO) 2024 priority pathogen report (World Health Organization, 2024).

Novel antimicrobial agents, including ceftazidime-avibactam (CZA), ceftolozane-tazobactam (CT), meropenem-vaborbactam (MEV), and imipenem-relebactam (IMP-REL), have been developed to address infections caused by multidrug-resistant (MDR) *P. aeruginosa*. IMP-REL combines a carbapenem with a non-*β*-lactam β-lactamase inhibitor active against Ambler class A and C *β*-lactamases. IMP-REL combines a carbapenem with a non-*β*-lactam β-lactamase inhibitor active against Ambler class A and C β-lactamases. Relebactam is a novel β-lactamase inhibitor that restores imipenem activity by inhibiting several *β*-lactamases, including Ambler class A enzymes (e.g., KPC) and class C enzymes (e.g., AmpC), but lacks activity against class B metallo-β-lactamases (Hilbert et al., 2023). Based on results from the SMART global surveillance program, relebactam has been shown to restore imipenem susceptibility among nonsusceptible isolates of *P. aeruginosa* and Enterobacterales, and to enhanced susceptibility among isolates with chromosomal AmpC production (Hilbert et al., 2023; Fraile-Ribot et al., 2020; Gomis-Font et al., 2020; Sader et al., 2023).

Previous *in vitro* studies have demonstrate that IMP-REL retains substantial activity against CRPA isolates, with higher susceptibility rates than imipenem alone across diverse geographic isolate collections. This activity is generally preserved in isolates with porin loss or efflux-mediated resistance, whereas reduced susceptibility is primarily associated with class B metallo-*β*-lactamases (MBLs) production (Hilbert et al., 2023; Fraile-Ribot et al., 2020; Sader et al., 2021). However, the impact of baseline carbapenem MICs, total resistance genes burden, and detailed β-lactamase class distribution on IMP-REL activity remains incompletely defined, particularly in high resistance settings. The present study addresses this gap by combining comprehensive phenotypic MIC testing with whole-genome sequencing.

At the time of manuscript submission, no studies were identified in regional or international databases focusing on IMP-REL susceptibility among CRPA isolates from Saudi Arabia or the MENA region. Therefore, this study aimed to evaluate the *in vitro* activity of IMP-REL against CRPA isolates and to assess its association with carbapenem susceptibility patterns and key *β*-lactamase–mediated resistance determinant.

## 2 Materials and methods

### 2.1 Study design and bacterial isolates

This retrospective, observational, analytical, laboratory-based study evaluated the in vitro activity of IMP-REL against CRPA. A total of 100 non-duplicate CRPA clinical isolates were collected between January 2023 and December 2024 at King Saud University Medical City in Riyadh, Saudi Arabia. Isolates were recovered from routine diagnostic specimens, including respiratory, blood cultures, wounds, urine, and body fluids of synovial, peritoneal, and pleural specimens. Only the first isolate per patient was included.

### 2.2 Study population

One hundred non-duplicate CRPA isolates were initially identified during the study period. Antimicrobial susceptibility testing was successfully completed for 99 isolates, while one isolate was excluded due to incomplete MIC data. Consequently, IMP-REL MICs were determined by the E-test method for 99 isolates, which constituted the final phenotypic analysis cohort.

Whole-genome sequencing (WGS) was performed on a subset of 66 isolates. These isolates were selected based on isolate availability and DNA quality, while ensuring representation across the observed IMP-REL susceptibility categories. The remaining isolates were not sequenced due to technical and logistical constraints.

### 2.3 Antimicrobial susceptibility testing (AST)

Bacterial identification was primarily performed using matrix-assisted laser desorption/ionization time-of-flight mass spectrometry (MALDI-TOF MS) (VITEK MS, bioMérieux, France) for rapid and accurate species-level identification. The VITEK 2 automated system (bioMérieux, France) was additionally used for organism identification. Conventional microbiological characteristics, including colony morphology, Gram staining, and biochemical reactions, were reviewed when necessary by a clinical microbiologist for verification.

Antimicrobial susceptibility testing (AST) was performed using the VITEK 2 automated system (bioMérieux, Marcy-l’Étoile, France) according to the manufacturer’s instructions. For carbapenems (imipenem and meropenem) and imipenem–relebactam (IMP-REL), minimum inhibitory concentrations (MICs) were determined using gradient diffusion (E-test; bioMérieux, France) to obtain quantitative MIC values. Bacterial suspensions were prepared from overnight cultures and adjusted to a 0.5 McFarland standard, inoculated onto Mueller–Hinton agar, and incubated at 35 ± 2 °C for 18–24 h. MIC values were defined as the lowest concentration inhibiting visible bacterial growth.

Interpretation of MIC results was performed according to Clinical and Laboratory Standards Institute (CLSI) guidelines (CLSI M100, current edition) (Clinical and Laboratory Standards Institute, 2024). For IMP-REL, isolates were categorized as susceptible (≤2 mg/L), intermediate (4 mg/L), or resistant (≥8 mg/L), with corresponding CLSI breakpoints applied for imipenem and meropenem. MIC₅₀ and MIC₉₀ values were calculated for all tested antimicrobial agents.

Quality control procedures were performed using *P. aeruginosa* ATCC 27853, and results were considered acceptable only when MIC values fell within established CLSI quality control ranges. The VITEK 2 system is implemented under College of American Pathologists (CAP) accreditation requirements, with routine internal validation and corrective actions undertaken when necessary.

Although broth microdilution is the reference standard for MIC determination, gradient diffusion methods (*E*-test) are widely used in routine clinical microbiology laboratories and have demonstrated acceptable categorical agreement with reference methods for carbapenem susceptibility testing in Gram-negative organisms (Kowalska-Krochmal and Dudek-Wicher, 2021; Wiegand et al., 2008). Disk diffusion and broth microdilution were not routinely performed as part of the clinical diagnostic workflow.

Phenotypic carbapenemase testing was not performed in this study, as resistance mechanisms were characterized using whole-genome sequencing, providing higher-resolution molecular data.

### 2.4 Whole-genome sequencing

Molecular resistance characterization was performed on a subset of 66 isolates, selected based on isolate availability and DNA quality; therefore, molecular classification results are reported exclusively for this subset. Genomic DNA was extracted from *P. aeruginosa* isolates using the QIAamp® DNA Mini Kit (QIAGEN, Germany) according to the manufacturer’s instructions. DNA quantity and purity were assessed prior to library preparation, following standardized protocols to ensure high-quality nucleic acid recovery suitable for whole-genome sequencing. Sequencing libraries were prepared using the QIAseq® FX DNA Library Kit and sequenced on the Illumina NextSeq platform with paired-end 2 × 150 bp reads, a configuration widely used for accurate bacterial genome reconstruction and resistance genes detection (QIAGEN, 2023). Raw sequencing reads were quality-checked and trimmed prior to *de novo* assembly and underwent stringent quality control, including assessment of base quality scores, GC content, duplication levels, and contamination, before downstream analysis. High-quality reads were assembled de novo using SPAdes and annotated with Prokka to identify coding sequences and functional genomic features (Petit III & Read, 2020). Antimicrobial resistance determinants were detected using AMRFinderPlus, and multilocus sequence typing and phylogenetic reconstruction were performed using the Bactopia pipeline, with Roary-based core-genome alignment and visualization in iTOL, enabling robust clonal and epidemiological inference (Seemann, 2014; Letunic & Bork, 2021). WGS was conducted using institutional laboratory resources without external funding support.

### 2.5 Statistical analysis

Descriptive statistics were used to summarize isolate characteristics, antimicrobial susceptibility results, and resistance mechanism profiles. Continuous variables, including minimum inhibitory concentration (MIC) values, were assessed for normality using visual inspection and the Shapiro–Wilk test and were found to be non-normally distributed. Accordingly, MICs are presented as medians with interquartile ranges (IQRs), whereas categorical variables are reported as frequencies and percentages.

MIC values for imipenem, meropenem, and imipenem–relebactam (IMP-REL) were analyzed as continuous variables on a log_2 scale to reflect the standard two-fold dilution scheme used in antimicrobial susceptibility testing. MIC {50} and MIC {90} values were calculated descriptively.

The relationship between IMP-REL MICs and comparator carbapenem MICs (imipenem and meropenem) was evaluated using Spearman’s rank correlation coefficient (\rho), given the non-normal distribution of MIC data.

Associations between IMP-REL MIC distributions and resistance determinants identified by whole-genome sequencing were analyzed separately. For binary variables, differences in MIC distributions were compared using the Mann–Whitney *U* test. For variables with more than two categories, the Kruskal–Wallis test was used. Resistance determinants with little or no variability across isolates, including universally detected mechanisms, were not subjected to formal comparative testing because such analyses would not be informative.

All tests were two-tailed, and a *p* value of < 0.05 was considered statistically significant. Statistical analyses were performed using SAS software, version 9.4 (SAS Institute Inc., Cary, NC, United States).

### 2.6 Ethical approval

This study was approved by the Institutional Review Board of King Saud University Medical City, Riyadh, Saudi Arabia (IRB approval number E-25-9914). The requirement for informed consent was waived due to the retrospective nature of the study and the use of anonymized bacterial isolates (Seemann, 2014). All procedures were conducted in accordance with institutional ethical standards and applicable regulations. No patient identifiers were collected, and strict confidentiality safeguards were maintained throughout data handling and analysis.

## 3 Results

**Figure 1:**
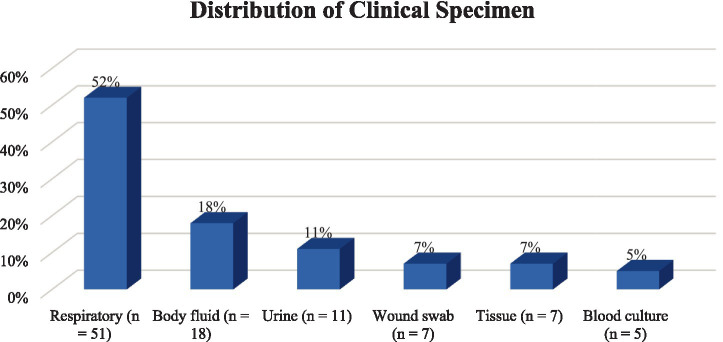
Distribution of clinical specimens among carbapenem-*resistant Pseudomonas aeruginosa* Isolates (*n* = 99).

A total of 99 non-duplicate CRPA isolates were included in the final phenotypic analysis. Most isolates were obtained from respiratory specimens (*n* = 51), followed by body fluid of synovial, peritoneal, and pleural specimens (*n* = 18), urine specimens (*n* = 11), wound swab specimens (*n* = 7), tissue specimens (*n* = 7), and blood culture specimens (*n* = 5). (Figure 1).

### 3.1 Antimicrobial susceptibility to IMP-REL

IMP-REL MIC values ranged from 0.38 to 32 mg/L, with a median MIC of 1.5 mg/L. The distribution demonstrated clustering at lower MIC values, with a smaller proportion of isolates exhibiting higher MICs (≥8 mg/L), forming a distinct resistant tail.

**Figure 2:**
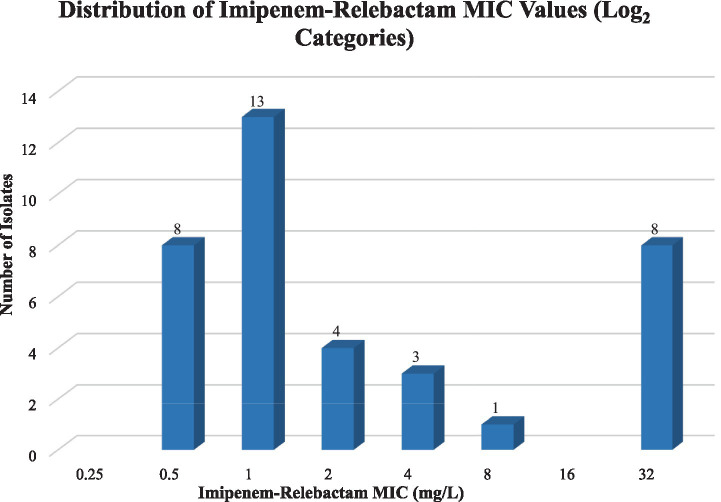
Distribution of imipenem–relebactam (IMP-REL) MIC values among carbapenem-resistant *Pseudomonas aeruginosa* isolates. Distribution of imipenem-relebactam (IMP-REL) MIC values among carbapenem-resistant *Pseudomonas aeruginosa* isolates. MIC values are presented on a log₂ scale. The distribution demonstrates clustering at lower concentrations (0.5–1.5 mg/L) with a distinct resistant tail at higher MICs (≥8 mg/L). One isolate was excluded due to missing MIC data.

The calculated MIC_50_ and MIC_90_ values for IMP-REL were 1.5 mg/L and 12 mg/L, respectively, indicating preserved *in vitro* activity against the majority of CRPA isolates, with a distinct subset demonstrating reduced susceptibility. (See Figure 2).

### 3.2 CLSI interpretive categories

**Figure 3:**
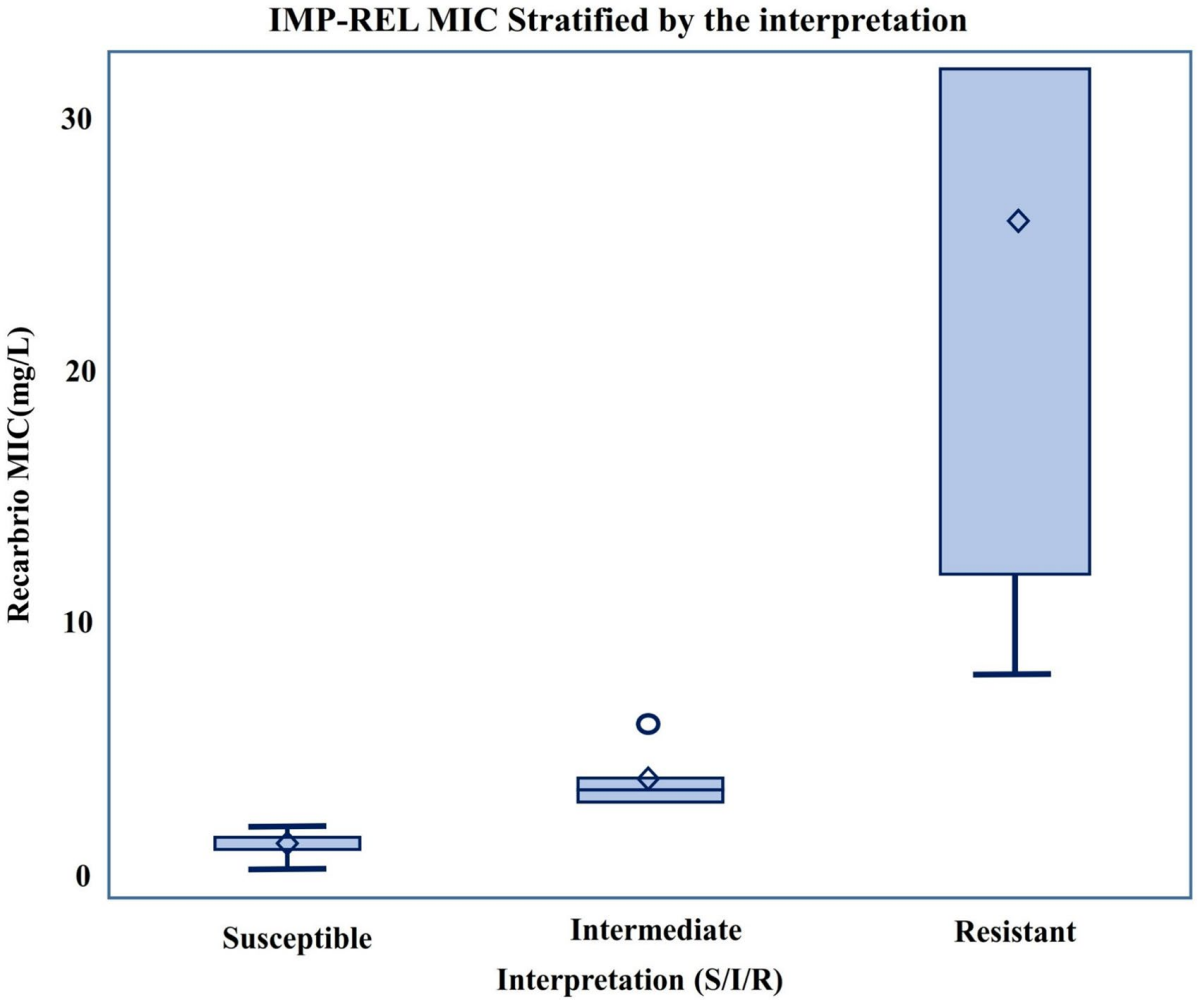
Distribution of imipenem–relebactam (IMP-REL) MIC values stratified by CLSI interpretive categories. MIC values are shown for susceptible, intermediate, and resistant isolates. Boxes represent the interquartile range (IQR), horizontal lines indicate the median, and whiskers represent the range of values. A stepwise increase in MIC values is observed across categories, consistent with CLSI classification.

Based on CLSI interpretive criteria, 78 isolates (78.8%) were classified as susceptible to IMP-REL, 10 (10.1%) as intermediate, and 11 (11.1%) as resistant. A stepwise increase in MIC values was observed across susceptibility categories, demonstrating consistency between quantitative MIC distributions and categorical interpretation (see Figure 3).

### 3.3 Correlation with other carbapenems

**Figure 4:**
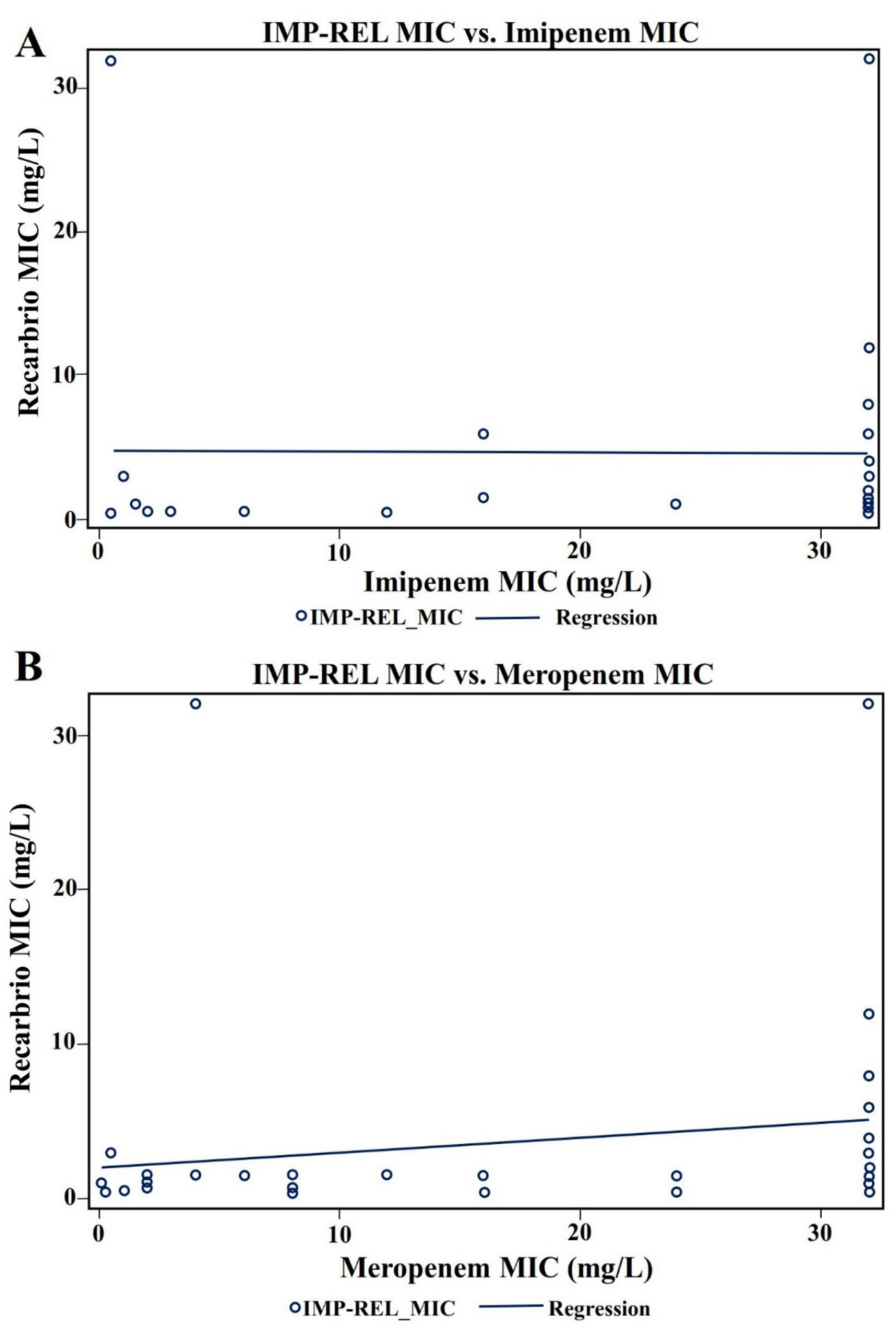
**(A)** Correlation between IMP-REL MIC and imipenem MIC. Footnote: Scatter plot illustrating the relationship between imipenem-relebactam (IMP-REL) minimum inhibitory concentrations (MICs) and imipenem MICs among carbapenem-resistant *P. aeruginosa* isolates. Spearman’s rank correlation demonstrated a weak but statistically significant positive association (*ρ* = 0.21, *p* = 0.041), while Pearson correlation showed no significant linear relationship. **(B)** Correlation between IMP-REL MIC and meropenem MIC. Footnote: Scatter plot showing the association between imipenem-relebactam (IMP-REL) minimum inhibitory concentrations (MICs) and meropenem MICs. A weak but statistically significant positive monotonic association was observed using Spearman’s rank correlation (*ρ* = 0.27, *p* = 0.0077), indicating that higher meropenem MIC ranks were associated with higher IMP-REL MICs despite the absence of a strong linear relationship.

Correlation analyses were performed to assess the relationship between IMP-REL MICs and MICs of comparator carbapenems. Spearman’s rank correlation demonstrated a weak but statistically significant monotonic association between IMP-REL and imipenem MICs (*ρ* = 0.21, *p* = 0.041) (see Figure 4A).

Similarly, IMP-REL MICs showed a significant positive association with meropenem MICs using Spearman’s rank correlation (*ρ* = 0.27, *p* = 0.0077) (see Figure 4B).

These findings suggest that increasing carbapenem MICs were associated with higher IMP-REL MICs in rank-based analyses.

### 3.4 Resistance mechanism profile

(Of the 99 CRPA) Isolates included in phenotypic susceptibility testing, WGS was successfully performed on 66 isolates, based on isolate availability and DNA quality. The remaining isolates were not subjected to molecular analysis and therefore contributed only to phenotypic susceptibility results. Accordingly, all resistance mechanisms and molecular classification analyses described in this section are based exclusively on the 66 sequenced isolates.

Resistance mechanism analysis of these 66 CRPA isolates revealed marked heterogeneity.

Class C (AmpC) *β*-lactamases were detected in 48 of the sequenced isolates (48/66, 72.7%), while Class D (OXA-type) *β*-lactamases were detected in all sequenced isolates (66/66, 100%), and efflux-associated resistance determinants were likewise universally present (66/66, 100%). In contrast, Class A *β*-lactamases were identified in 9 isolates (13.6%), while MBLs (Class B) were detected in 5 isolates (7.6%). Multiple resistance mechanisms were frequently co-expressed, highlighting the substantial molecular resistance complexity within the sequenced CRPA subset (see Table 1).

**Table 1:** Number and percentage of resistance classes (*n* = 66).

Resistance mechanism	Number (*n*)	Percentage (%)	Genes detected
Class A	9	13.6%	VEB_1b, VEB_9 and GES_4
Class B	5	7.6%	IMP_34 and VIM_28
Class C	48	72.7%	PDC_1, 2, 3, 5, 7, 8, 10 and DHA_1
Class D	66	100.0%	OXA_10, 50, 232, 485, 486, 488 and LCR_1
Efflux	66	100.0%	MexA, B, C, D, E, F MuxA, B, C OpmB, H, J, M, N

Resistance mechanisms were identified by whole-genome sequencing in a subset of 66 carbapenem-resistant *Pseudomonas aeruginosa* isolates. Class D (OXA-type) *β*-lactamases, as well as efflux-associated resistance determinants, were detected in all sequenced isolates (100%), precluding correlation analysis for these mechanisms. Class C (AmpC-type) *β*-lactamases were identified in 48 isolates (72.7%), while Class A and Class B *β*-lactamases were detected less frequently and are presented as the proportion of isolates harboring at least one gene belonging to each resistance class.

At the individual gene level, OXA-486 was the most prevalent *β*-lactamase, detected in 35 of 66 isolates (53%), followed by OXA-50 and OXA-488 (22.7% each). Among AmpC enzymes, PDC-3 (18.2%) and PDC-5 (15%) were the most frequently identified variants. Class A *β*-lactamases were less common, with VEB-9 detected in 7.6% of isolates. Metallo-*β*-lactamases were infrequent, with IMP-34 identified in 6% and VIM-28 in 1.5% of isolates. (See Supplementary Table S1).

### 3.5 Correlation between IMP-REL MIC and resistance mechanisms (*n* = 66)

Spearman rank correlation analysis demonstrated weak positive correlations between IMP-REL MICs and the presence of Class A (*ρ* = 0.23) and Class B (*ρ* = 0.23) resistance mechanisms. Additionally, weak but statistically significant correlations were observed between IMP-REL MICs and carbapenem MICs.

No meaningful correlations were identified between IMP-REL MICs and other resistance determinants. It should be noted that resistance mechanisms lacking variability (e.g., Class C, Class D *β*-lactamases, and efflux-associated determinants), which were universally present among the analyzed isolates, were not included in the correlation analysis, as such comparisons are not statistically informative (see Figure 4B).

## 4 Discussion

This study demonstrates substantial *in vitro* activity of IMP-REL against a genetically diverse population of CRPA. Overall, 78.8% of *P. aeruginosa* isolates demonstrated susceptibility when relebactam was added to imipenem. Despite elevated carbapenem MICs and the presence of multiple resistance genes, IMP-REL MIC values remained relatively low and consistent across isolates. These findings support previous surveillance reports indicating preserved activity of IMP-REL against isolates harboring heterogeneous resistance mechanisms (Horner et al., 2019).

Similar observations have been reported in multicenter studies evaluating IMP-REL against CRPA, where susceptibility rates varied according to the prevalence of MBLs (Lob et al., 2020). In the SMART surveillance program (2015–2017), 79.7% of MDR *P. aeruginosa* isolates from intra-abdominal infections and urinary tract infections were susceptible to IMP-REL, compared with only 33.3% susceptible to imipenem alone (Lob et al., 2020). Likewise, in the CANWARD surveillance study (2016–2019), the addition of relebactam enhanced the in vitro activity of imipenem against 70.8% of MDR *P. aeruginosa* clinical isolates (Horner et al., 2019; Walkty et al., 2021).

A distinguishing feature of the present study is its focus on a regional collection of carbapenem-resistant *P. aeruginosa*, combined with detailed analysis of carbapenem MIC distributions and *β*-lactamase profiles. This design enabled a more granular assessment of how specific resistance determinants influence the activity of IMP-REL. Given the limited data describing the activity of IMP-REL against CRPA in the Middle East remain limited, our findings provide important regional microbiological evidence that may help guide antimicrobial stewardship strategies and therapeutic decision-making in settings with similar resistance epidemiology.

The activity of IMP-REL in our study was not substantially affected by conventional carbapenem resistance mechanisms, as only weak but statistically significant correlations were observed between IMP-REL MICs and carbapenem MIC values, indicating limited strength of association. IMP-REL activity appeared largely preserved despite the widespread presence of Class C (AmpC) and Class D (OXA-type) β-lactamases, likely reflecting the limited impact of these intrinsic mechanisms on IMP-REL activity. Efflux-associated resistance determinants were also universally present and did not appear to significantly influence IMP-REL MIC values. In contrast, Class B metallo-β-lactamases (MBLs), although infrequent, were associated with higher IMP-REL MIC values, consistent with the known lack of relebactam activity against these enzymes. Class A β-lactamases demonstrated weak positive associations with IMP-REL MICs, although their overall prevalence was limited in this cohort. These findings are consistent with large surveillance studies demonstrating that the addition of relebactam significantly reduces imipenem MICs by several dilution steps, regardless of efflux pump overexpression or chromosomal AmpC β-lactamase activity (Horner et al., 2019).

In our dataset, class B carbapenemases MBLs were underrepresented, which may partially explain the relatively high susceptibility rates observed. Previous studies have shown that *P. aeruginosa* isolates harboring class B MBLs typically exhibit elevated IMP-REL MICs because relebactam does not inhibit these enzymes. Consequently, cohorts with higher MBLs prevalence would be expected to demonstrate lower susceptibility rates (Young et al., 2019).

Clinical and surveillance data further support the broad *in vitro* activity of IMP-REL against carbapenem-resistant gram-negative pathogens. Several studies have reported IMP-REL susceptibility rate of approaching 84%, compared with 56% for imipenem alone, highlighting the substantial activity provided by the addition of relebactam against carbapenem-resistant isolates (Zhang et al., 2021). In the clinical setting, the RESTORE IMI-1 randomized, double-blind trial compared IMP-REL with colistin plus imipenem in patients with imipenem-non-susceptible gram-negative infections, many of which were *P. aeruginosa*. IMP-REL demonstrated comparable overall clinical response rates (approximately 70%) and showed trends toward lower mortality and reduced nephrotoxicity compared with colistin-based therapy (Motsch et al., 2020).

Taken together, these findings highlight the potential role of IMP-REL as an important therapeutic option for CRPA. From a clinical perspective, the integration of IMP-REL into antimicrobial stewardship programs may facilitate optimized treatment strategies by preserving its activity against isolates harboring multiple resistance mechanisms while limiting unnecessary exposure nephrotoxic salvage alternatives such as polymyxins.

A Major strength of this study is the integration of phenotypic susceptibility testing with molecular characterization of resistance determinants, providing comprehensive assessment of IMP-REL activity against genetically diverse CRPA isolates.

The resistance mechanisms of *P. aeruginosa* are complex and multifactorial. Efflux pump overexpression and porin downregulation (particularly OprD loss) play major roles in carbapenem resistance (Xavier et al., 2010). In the present study, expression levels of these resistance determinants were not evaluated. However, recent mechanistic studies suggest that imipenem may partially bypass OprD-mediated resistance through alternative entry pathways and may induce the expression of chromosomal AmpC β-lactamase, thereby creating a functional target for inhibition by relebactam (Freed Jr & Hanson, 2024). Future studies incorporating functional analyses- such as gene-expression and permeability assays- may provide further insight into the interaction between resistance mechanisms and IMP-REL susceptibility.

Finally, this study was conducted at a single center and included relatively limited number of isolates, which may restrict the generalizability of the findings to other epidemiologic setting. Larger multicenter and regional studies are therefore warranted to validate these observations and better define the role of IMP-REL in the management of CRPA infections.

## 5 Conclusion

IMP-REL demonstrated substantial in vitro activity against CRPA, with susceptibility observed in the majority of isolates despite complex resistance profiles. These findings support its potential role as an effective therapeutic option for CRPA infections and provide important regional data from the Middle East, where such evidence remains limited. Integration of IMP-REL into antimicrobial stewardship strategies may optimize treatment while reducing reliance on more toxic alternatives. Further multicenter studies are warranted to validate these findings across diverse epidemiologic settings.

## Data Availability

The raw data supporting the conclusions of this article will be made available by the authors, without undue reservation.
