# Supplementary material for: In vitro activity of imipenem–relebactam against carbapenem-resistant Pseudomonas aeruginosa: correlation with carbapenem MICs and association with β-lactamase classes
Source: Front Microbiol. 2026 May 7;17:1790477. doi: 10.3389/fmicb.2026.1790477 (PMC13190585; doi:10.3389/fmicb.2026.1790477)
Supplement: Supplementary file 1 [file Table_1.docx]

# **Supplementary Table 1. Frequency of individual resistance genes among sequenced CRPA isolates (n = 66)**

| Resistance Class | Gene | No. of Isolates (n) | Frequency (%) |
| --- | --- | --- | --- |
| Class A | VEB-1b | 1 | 1.5 |
|  | VEB-9 | 5 | 7.6 |
|  | GES-14 | 3 | 4.5 |
| Class B (MBL) | IMP-34 | 4 | 6 |
|  | VIM-28 | 1 | 1.5 |
| Class C (AmpC) | PDC-1 | 6 | 9 |
|  | PDC-2 | 9 | 13.6 |
|  | PDC-3 | 12 | 18.2 |
|  | PDC-5 | 10 | 15 |
|  | PDC-7 | 2 | 3 |
|  | PDC-8 | 3 | 4.5 |
|  | PDC-10 | 6 | 9 |
|  | DHA-1 | 1 | 1.5 |
| Class D (OXA-type) | OXA-10 | 4 | 6 |
|  | OXA-50 | 15 | 22.7 |
|  | OXA-232 | 2 | 3 |
|  | OXA-485 | 1 | 1.5 |
|  | OXA-486 | 35 | 53 |
|  | OXA-488 | 15 | 22.7 |
| Efflux Pumps | MexA | 66 | 100 |
|  | MexB | 66 | 100 |
|  | MexC | 66 | 100 |
|  | MexD | 66 | 100 |
|  | MexE | 66 | 100 |
|  | MexF | 66 | 100 |
|  | MuxA | 66 | 100 |
|  | MuxB | 66 | 100 |
|  | MuxC | 66 | 100 |
|  | OpmB | 66 | 100 |
|  | OpmH | 66 | 100 |
|  | OprJ | 66 | 100 |
|  | OprM | 66 | 100 |
|  | OprN | 66 | 100 |
